# Synthesis and characterizations of YZ-BDC:Eu^3+^,Tb^3+^ nanothermometers for luminescence-based temperature sensing

**DOI:** 10.1039/d2ra01759h

**Published:** 2022-04-29

**Authors:** Lam Thi Kieu Giang, Karolina Trejgis, Łukasz Marciniak, Agnieszka Opalińska, Iwona E. Koltsov, Witold Łojkowski

**Affiliations:** Institute of Materials Science, Vietnam Academy of Science and Technology 18 Hoang Quoc Viet Cau Giay Hanoi Viet Nam giangltk@ims.vast.ac.vn; Graduate University of Science and Technology, Vietnam Academy of Science and Technology 18, Hoang Quoc Viet Cau Giay Hanoi Viet Nam; Institute of Low Temperature and Structural Research, Polish Academy of Sciences Okólna 2 50-422 Wrocław Poland; Institute of High Pressure Physics, Polish Academy of Sciences Sokolowska Street 29/37 Warsaw Poland

## Abstract

In the present work, nanothermometers based on amorphous zirconium metal–organic frameworks co-doped with rare-earth ions (YZ-BDC:Eu^3+^,Tb^3+^ nanothermometers) with sizes of about 10–30 nm were successfully synthesized *via* a microwave-assisted hydrothermal method at 120 °C for 15 min. The determined BET surfaces area, total pore volume and average pore diameter were ∼530 m^2^ g^−1^, 0.45 cm^3^ g^−1^ and 3.4 nm, respectively. Based on Fourier transform infrared spectroscopy (FTIR) and simultaneous thermal analysis (STA) results, the formation process of carboxylic acid salts and the molecular formula of the samples have been proposed. The thermometric properties of Zr-BDC:Eu^3+^,Tb^3+^ nanothermometers and their Y^3+^ ion co-doped counterparts (YZ-BDC:Eu^3+^,Tb^3+^) measured in the 133–573 K temperature range were compared. Moreover, the temperature-dependent CIE(x, y) chromaticity coordinates and emission color of the samples were also determined. As the temperature increased from 133 to 573 K, the emission color of Zr-BDC:Eu^3+^,Tb^3+^ nanothermometers without the presence of Y^3+^ ions changed from orange to red, while for YZ-BDC:Eu^3+^,Tb^3+^ nanothermometers, the emission color changed from yellow to orange, due to the strong effect of the presence of Y^3+^ ions on the luminescence intensity of Eu^3+^ and Tb^3+^ ions. The maximum relative sensitivity (*S*_Rmax_) in both materials was close to 0.5%/K, however, the temperature range of their occurrence was significantly shifted toward higher temperatures due to doping with Y^3+^ ions. The obtained results showed that doping with Y^3+^ ions not only enables the modulation of the useful temperature range with high relative sensitivity, but also provides improved thermal stability.

## Abstract 2

In the present work, nanothermometers based on amorphous zirconium metal–organic frameworks co-doped with rare-earth ions (YZ-BDC:Eu^3+^,Tb^3+^) with sizes of about 10–30 nm were successfully synthesized via a microwave-assisted hydrothermal method at 120 °C for 15 min.

## Graphical abstract

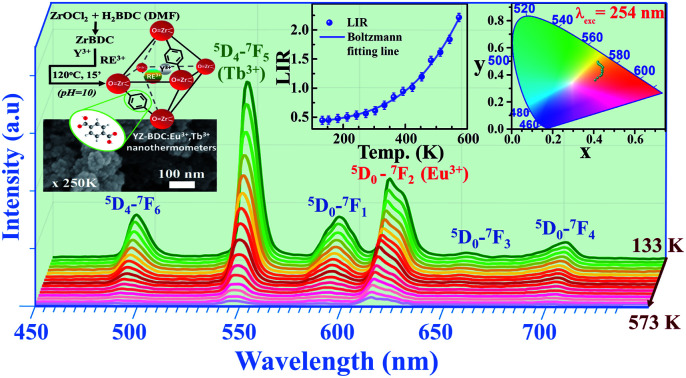

## 1. Introduction

Temperature is one of the most crucial physical parameters for understanding or determining the occurrence of certain processes in technological or biological systems. Thus, it is of interest to many research groups in various scientific fields and research on ideal temperature measurement devices has become a hot topic.^1–5^ Temperature measuring devices can be divided into two main groups *i.e.* contact and noncontact thermometers. In addition to thermovision cameras, noncontact thermometry includes luminescence thermometry, which is an unconventional technique that has attracted the interest of many researchers due to a number of distinguishing features such as high temperature resolution, high relative thermal sensitivity, short acquisition times, and the ability to accurately determine the temperature of objects in various environments without any physical contact, such as biological fluids, fast moving objects, strong electromagnetic fields, *etc.*^1–6^ Using the luminescence thermometry technique, the temperature is not measured directly, but determined from thermally induced changes in spectroscopic properties. In general, there are several classes of luminescence thermometers (LTs) in which the temperature-dependent parameter may be: luminescence intensity ratio (LIR), lifetime, band shift, bandwidth, *etc.*^7,8^ In the search for an optimal thermometer, not only various thermometric techniques are analyzed but also a range of materials are explored, with the rare earth inorganic compounds,^1,2,6,8^ carbon dots (CDs),^3^ organic molecules,^9^ polymers^10^ and metal–organic framework (MOF) materials^11–13^ leading the way.

Although MOF materials have the disadvantages of low stability and conductivity, as well as the leaching effect of metals in liquids, they are characterized by substantial advantages in terms of controlling the composition of dopant, morphology, size and structure, as well as the dispersion and biocompatibility, *etc.*^13–15^ Therefore, research towards improving the stability and durability of MOF-based thermometers is still one of the most important issues in order to expand and optimize the possibilities of their applications in the field of temperature sensors, in particular, temperature measurements of biological organisms. Therefore, the aim of this work was to prepare a MOF material that would simultaneously be characterised by size on the nanometric scale and yet exhibit high thermal stability, chemical durability, good dispersion properties and stability in water and organic solvents. Furthermore, the aim of the work was to prepare a material that would also exhibit suitable luminescent properties, with potential application in optical thermometry.

Among MOF materials that are stable in water or organic solvents, those that are formed by binding Zr(iv) and carboxylate ligands to form Zr-MOF are particularly notable. The high charge density, bond polarization as well as strong affinity make this material stable not only in water and organic solvents, but even in acidic aqueous solutions.^16,17^ Unfortunately, many types of Zr-MOFs formed by zirconium-carboxylate linkers are characterized by sizes in the range of tens of nm to tens of μm. These include UiO-66, MOF 804, MIL-140A, Zr-ABDC, *etc.*^16–21^ However, for many applications, especially biomedical applications such as temperature sensing and imaging, the size of Zr-MOF thermometers must be reduced to the nanoscale.^22^ One of the most effective solutions to reduce the size of these thermometers is to use an appropriate amount of H_2_BDC and adjust the pH of the zirconium carboxylate precursor solutions, as well as to conduct microwave-assisted hydrothermal reactions.^23^ In this work, NH_4_OH solution was used to adjust pH of the zirconium carboxylate precursors solutions and the synthesis were carried out in the microwave-assisted reaction system at 120 °C (393 K) for 15 min with pressure reaching up to 6 at. The zirconium metal–organic frameworks synthesized in this way had amorphous structures with sizes of about 10–30 nm. Furthermore, it is shown in this work that the thermal stability and sensitivity of YZ-BDC:Eu^3+^,Tb^3+^ nanothermometers can be improved by adjusting the amount of Y^3+^ ions in the samples.

## 2. Experimental

### 2.1. Materials

Zirconium(iv) oxychloride octahydrate (ZrOCl_2_·8H_2_O, 98%), europium chloride hexahydrate (EuCl_3_·6H_2_O, 99.9%), terbium chloride hexahydrate (TbCl_3_·6H_2_O, 99.9%), yttrium chloride hexahydrate (YCl_3_·6H_2_O, 99.9%), terephthalic acid (H_2_BDC or C_6_H_4_(COOH)_2_, 98%), dimethylformamide (DMF or HCON(CH_3_)_2_, 99.8%), ammonium hydroxide solution (NH_4_OH) and ethanol (CH_3_CH_2_OH, 99.5%) were purchased from Sigma Aldrich.

### 2.2. Synthesis methods

The YZ-BDC:Eu^3+^,Tb^3+^ nanothermometers were prepared by soft-template method as follows:

Initially, 1.16 g (3.6 mmol) ZrOCl_2_·8H_2_O salt was dissolved in a glass vial with 30 ml of mixture of water (H_2_O) and methanol (volume ratio of 1 : 2) in an ultrasonic bath for 10 min and then stirred at 30 °C for 20 min. Simultaneously, 0.83 g (5 mmol) of H_2_BDC in another glass vial containing 30 mL of DMF was dispersed in an ultrasonic bath for 20 min and then added at 30 °C to the magnetically stirred solution prepared in the first step and maintained for 2 hours to obtain a homogeneous solution A.

Then, a rare earth mixture solution containing 0.6 ml of YCl_3_ (0.15 mmol, 0.25 M), 1 mL of EuCl_3_ (0.25 mmol, 0.25 M), 4 mL of TbCl_3_ (1 mmol, 0.25 M) and 20 mL of methanol was vigorously stirred in a glass vial for 20 min, after which it was added dropwise to the magnetically stirred previously prepared solution A to obtain a homogeneous complex solution B.

Finally, using NH_4_OH 25%, the pH of complex solution B was adjusted to 10 and the solution was poured into a Teflon vessel of the microwave reactor. The synthesis reaction was carried out in the Magnum II Microwave reactor (ERTEC, IHPP PAS, Poland) at 120 °C for 15 min (pressure reached up to 6 at) and cooled to room temperature for 20 min. Finally, the precipitation products were filtered, soaked in DMF solution for 12 hours and then washed by centrifugation three times with DMF and three times with methanol to obtain the white powder of YZ-BDC:Eu^3+^,Tb^3+^ nanothermometers.

### 2.3. Characterization

The surface morphology characteristics of the synthesized YZ-BDC:Eu^3+^,Tb^3+^ nanothermometers was observed by scanning electron microscope (SEM, Ultra Plus, Zeiss, Oberkochen, Germany). The structural analysis of the samples was performed by X-ray diffraction (X'Pert PRO, PANalytical, Almelo, Netherlands) using a Cu*K*α_1_ copper anode in the 2*θ* range from 5° to 100° with the ultrafast step of 0.03°. The Brunauer–Emmett–Teller (BET) surface area and pore size analysis were determined from adsorption–desorption isotherms, which were performed using a micrometer instrument (Tristar 3000 V6.07 A) at 77 K. Samples were degassed at 423 K (150 °C) for 3 h prior to measurement. The total pore volume was calculated for the highest relative pressure point (*P*/*P*_0_ = 0.99).

FTIR analysis was performed using a FT-IR-spectrometer (Bruker Tensor 27, Germany) in the spectral range from 4000 to 400 cm^−1^. STA analysis consisting of thermogravimetry (TG) and differential scanning calorimetry (DSC) was carried out in helium flow in the temperature range from 297 to 1273 K using a Jupiter STA 449 F1 instrument (Netzsch, Selb, Germany) with a heating rate of 10 K min^−1^. The temperature-dependent emission spectra upon 254 nm excitation were collected over a wide temperature range from 133 to 573 K using a Jobin–Yvon HR1000 monochromator with a charge-coupled device (CCD) camera and THMS600 heating/cooling stage instrument (Linkam).

## 3. Result and discussion

### 3.1. The morphology and structure of the obtained YZ-BDC:Eu^3+^,Tb^3+^ nanothermometers

**Fig. 1:**
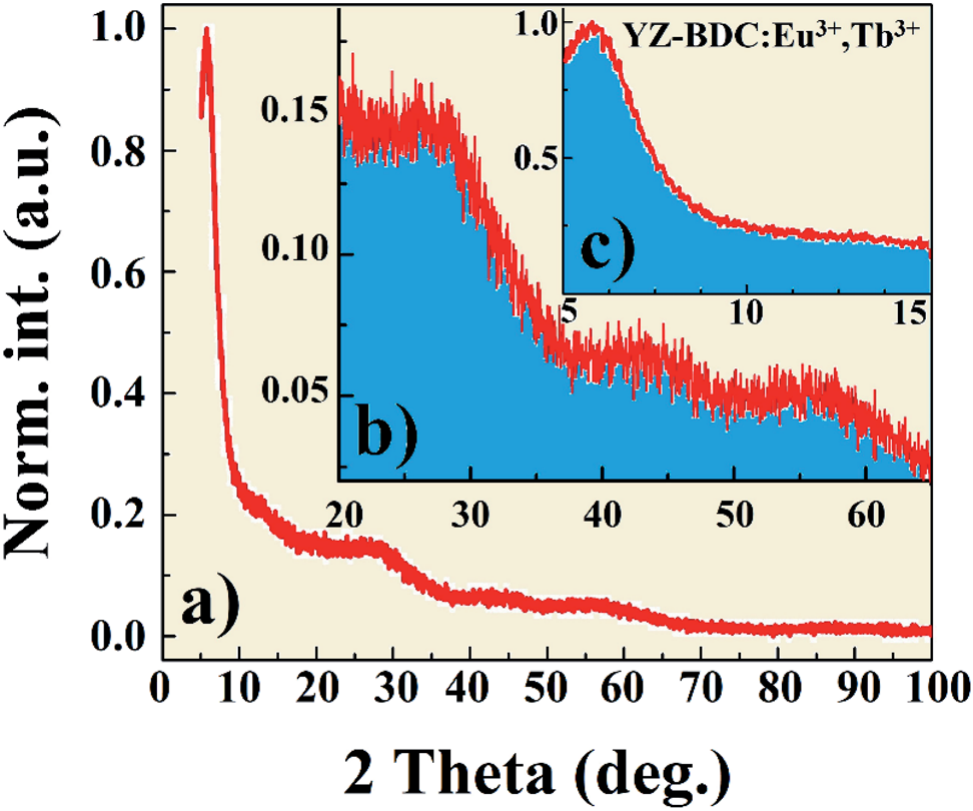
The X-ray diffraction pattern of the YZ-BDC:Eu^3+^,Tb^3+^ nanothermometers synthesized under closed microwave reactor conditions at 120 °C for 15 min at pressures up to 6 atm in the 2*θ* range from: 0° to 100° (a), and 20° to 65° (b), and 5° to 15° (c).

X-ray diffraction pattern of the YZ-BDC:Eu^3+^,Tb^3+^ nanothermometers synthesized under closed microwave reactor conditions at 120 °C for 15 min at pressures up to 6 atm is presented in Fig. 1. One sharp reflection peak 2*θ* ∼6° (Fig. a and c) and two broad reflection peaks with maxima around 2θ ∼27° and 55° were observed (Fig. 1a and b), which were assigned to the diffraction of amorphous zirconium metal–organic frameworks, which is consistent with the UiO-66 and MIL-140 (Zr) patterns previously reported.^19,24^

**Fig. 2:**
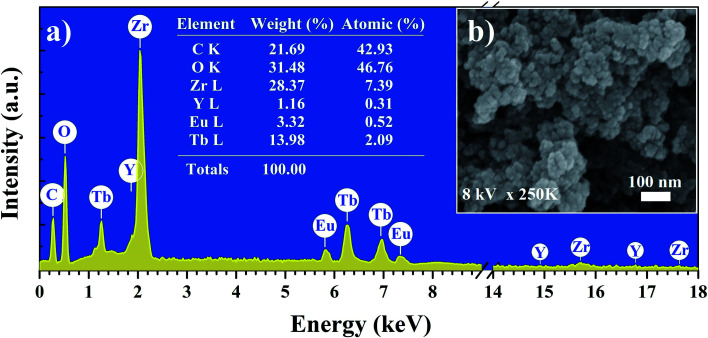
Elemental analysis (EDX) (a) and Scanning electron microscopy images (SEM) (b) of the YZ-BDC:Eu^3+^,Tb^3+^ nanothermometers.

Fig. 2 shows the SEM image and EDX analysis of the YZ-BDC:Eu^3+^,Tb^3+^ nanothermometers. Based on the analysis of SEM images (Fig. 2b) of YZ-BDC:Eu^3+^,Tb^3+^ nanothermometers, a porous particle structure and an average size of about 10–30 nm were found. Moreover, the presence of C, O, Zr, Y, Eu, and Tb elements in the obtained materials was confirmed by elemental analysis (Fig. 2a), whereby high weights of C (>20%) and O (>30%) elements indicate the existence of an organic structure.

**Fig. 3:**
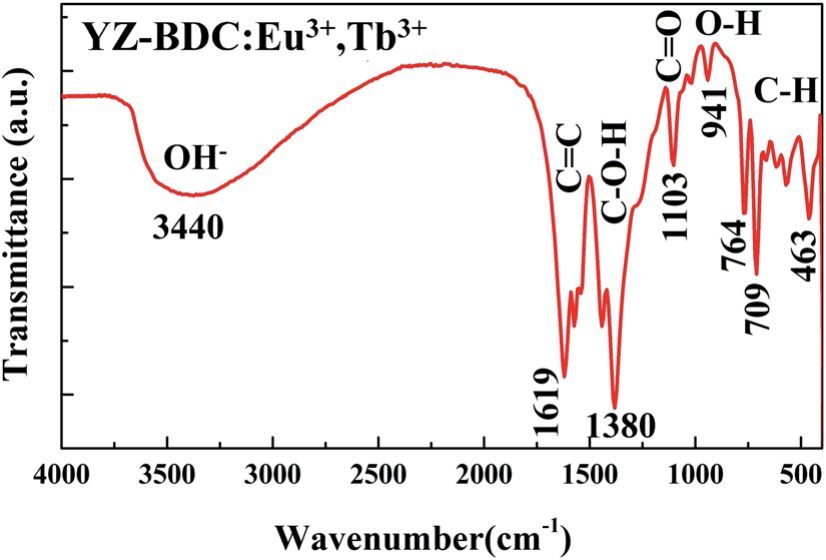
FTIR spectra of the YZ-BDC:Eu^3+^,Tb^3+^ nanothermometers in the wavenumber range from 4000 to 400 cm^−1^.

The formation of metal–organic framework structure in the YZ-BDC:Eu^3+^,Tb^3+^ nanothermometers was further confirmed by the FTIR analysis (Fig. 3).

The broad band in the region between 3700–2780c m^−1^ indicates the presence of stretching O–H interactions of carboxylic acids, as well as the possible presence of water molecules in samples.^19,25^ The absorbance peaks at around 1619, 1573 and 1542 cm^−1^ are associated to the C=C stretching vibration bands of unique aromatic ring compounds, which confirms the formation of the carboxylic acid salts. The absorbance peaks in the 1440–1380 cm^−1^ and 1265–1103 cm^−1^ region are due to the C–O–H and C=O bending vibrations indicating the presence of carboxylic acid groups in the samples. The weak absorbance peaks at around 1018 cm^−1^ due to presence of C–N stretching bands of DMF indicate that the DMF residues in the samples are very small. The absorbance peak at around 941 cm^−1^ is associated with out-of-plane O–H bending vibrations of carboxylic acid, which also indicates the presence of hydrated clusters. Furthermore, absorbance peaks below 900 cm^−1^ assigned to the out-of-plane C–H bands of the aromatic ring confirmed even more clearly the formation of the zirconium metal–organic frameworks compounds.^20,26^

### 3.2. The thermal stability of the YZ-BDC:Eu^3+^,Tb^3+^ nanothermometers

**Fig. 4:**
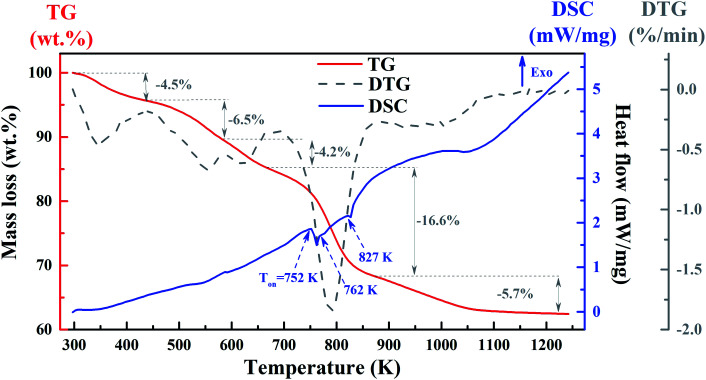
The TG (red line), DSC (blue line) and derivative of TG signal – DTG (dark line) curves of the YZ-BDC:Eu^3+^,Tb^3+^ nanothermometers.

The thermal analysis of the YZ-BDC:Eu^3+^,Tb^3+^ nanothermometers was investigated by TG and DSC (Fig. 4).

As can be seen from the TG curves (red line) in Fig. 4, the first mass loss of −4.5% recorded at temperatures ranging from 298 and 443 K (25–170 °C) is due to the removal of surface hydroxyl groups. The second mass loss of −6.5% at temperatures between 443 and 583 K (170–310 °C) corresponds to the removal of organic solvents. The third mass loss of −4.2% in the temperature range from 583 to 668 K (310–395 °C) is related to the process of dehydroxylation of zirconium oxy-clusters. This is consistent with the small endothermic peak at 587 K observed in the DSC curves (blue line) and the FTIR results obtained above. The largest mass loss of −16.6% at temperatures between 668 and 873 K (395–600 °C) and a final mass loss of −5.7% at temperatures above 873 K (600 °C) suggest decomposition YZ-BDC:Eu^3+^,Tb^3+^ nanothermometers from zirconium metal–organic framework to the ZrO_2_ form. This is consistent with a major endothermic peak at temperature onset (*T*_on_) at about 752 K (479 °C) and two minor endothermic peaks with *T*_on_ ≈ 762 K (489 °C) and 827 K (555 °C), as presented in the DSC curve (blue line). It also confirms the phase transformation from the amorphous zirconium metal–organic framework to YZ-BDC:Eu^3+^,Tb^3+^ nanocrystals.^19,27^

Considering all the results obtained, the formation mechanism of YZ-BDC:Eu^3+^,Tb^3+^ nanothermometers is proposed as follows:^21^

1 ZrOCl_2_ + C_6_H_4_(COOH)_2_ → ZrOC_6_H_4_(COO)_2_ + 2HCl

2 (1 − *x*)ZrOC_6_H_4_(COO)_2_ + *x*RE^3+^ → Zr_1−*x*_OC_6_H_4_(COO)_2_:RE^3+^_*x*_

3
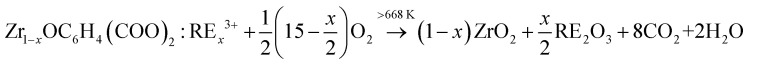


### 3.3. The nitrogen adsorption–desorption isotherms of the YZ-BDC:Eu^3+^,Tb^3+^ nanothermometers

**Fig. 5:**
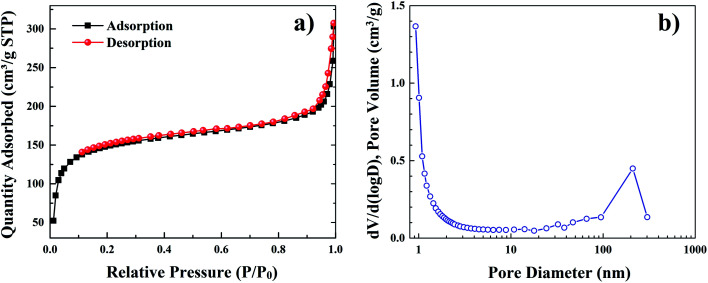
The nitrogen adsorption–desorption isotherms (a) and corresponding calculated pore size distribution (b) of YZ-BDC:Eu^3+^,Tb^3+^ nanothermometers.

The nitrogen adsorption–desorption isotherms measured at temperature of 77 K show a small hysteresis loop plateau at partial pressure *P*/*P*_0_ from 0.4 to 1.0, which is similar to the type I isotherms (Fig. 5a).

Based on these measurements, the pore size distribution of YZ-BDC:Eu^3+^,Tb^3+^ nanothermometers was calculated (Fig. 5b). The calculated values of multiple BET surfaces area (S_BET_), total pore volume (*V*_p_) and average pore diameter (*D̄*_p_) are 528.2 m^2^ g^−1^, 0.45 cm^3^ g^−1^, and 3.4 nm, respectively. This confirms the characteristic porosity of the YZ-BDC:Eu^3+^,Tb^3+^ structure.

### 3.4. The temperature-dependent emission spectra of the YZ-BDC:Eu^3+^,Tb^3+^ nanothermometers

**Fig. 6:**
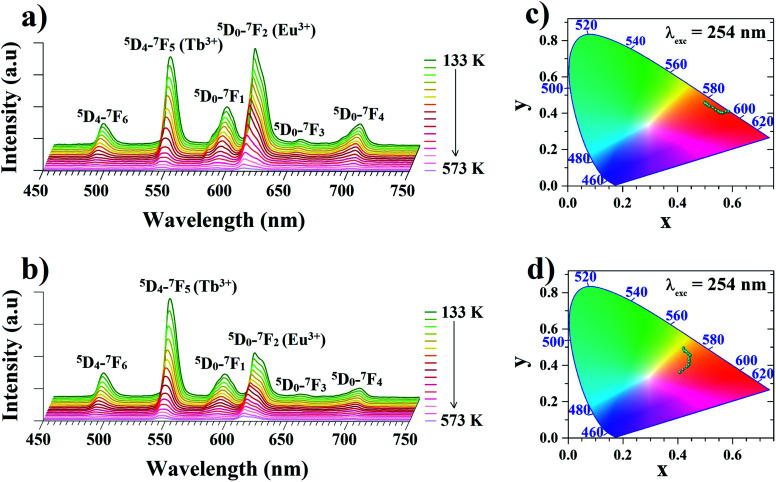
The temperature-dependent emission spectra of Zr-BDC:Eu^3+^,Tb^3+^ (a) and YZ-BDC:Eu^3+^,Tb^3+^ (b) nanothermometers recorded under the excitation of 254 nm in temperature range of 133–573 K and corresponding calculated CIE chromaticity diagrams (c and d, respectively).

In order to determine the suitability of the analyzed materials for luminescence thermometry, the thermal evolutions of emission spectra of the Zr-BDC:Eu^3+^,Tb^3+^ (without the presence of Y^3+^ ions) and the YZ-BDC:Eu^3+^,Tb^3+^ nanothermometers were measured upon 254 nm excitation in the temperature range from 133 to 573 K. In the emission spectra of analyzed materials (Fig. 6a and b), the presence of six emission peaks can be clearly observed, with the bands located at 491 and 545 nm originating from ^5^D_4_–^7^F_*J*_ (*J* = 6, 5) electronic transitions of Tb^3+^ ions, while the bands at 589, 614, 651 and 701 nm are associated with ^5^D_0_–^7^F_*J*_ (*J* = 1–4) electronic transitions of Eu^3+^ ions, respectively.

As can be seen from the comparison of the temperature-dependent emission spectra of both these materials, the presence of Y^3+^ ions in the host significantly affects the intensity of the particular bands. In the host without Y^3+^ doping, in the whole analyzed temperature range the dominant emission band in the spectrum is the band located at 614 nm originating from the luminescence of Eu^3+^ ions. In the YZ-BDC:Eu^3+^,Tb^3+^ material, on the other hand, it can be observed that the presence of the Y^3+^ dopant changes the relative intensity ratio of the bands of Tb^3+^ ions relative to Eu^3+^ ions and at low temperatures a significantly higher luminescence intensity is observed for the emission band at 545 nm associated with the luminescence of Tb^3+^ ions.

Due to the observed strong dependence of the intensity of particular emission bands on both the host composition and temperature, the CIE (*x*,*y*) values were calculated for both analyzed materials. With increasing temperature from 133 to 573 K the calculated CIE values of Zr-BDC:Eu^3+^,Tb^3+^ (without the presence of Y^3+^ ions) changed from (0.4990, 0.4600) to (0.5656, 0.4013) with the emission color change from orange to red (Fig. 6c and Table 1a). For materials doped with Y^3+^ ions (YZ-BDC:Eu^3+^,Tb^3+^), the calculated CIE (*x*, *y*) values changed from (0.4208, 0.4944) to (0.4079, 0.3604), which also demonstrated a gradual change in emission color from yellow to orange (see in Fig. 6d and Table 1b).

**Table 1:** The CIE chromaticity coordinates of samples: (a) Zr-BDC:Eu^3+^,Tb^3+^ and (b) YZ-BDC:Eu^3+^,Tb^3+^ nanothermometers

Samples	(a) Zr-BDC:Eu^3+^,Tb^3+^		(b) YZ-BDC:Eu^3+^,Tb^3+^	
Temperature (K)	CIE		CIE	
	*x*	*y*	*x*	*y*
133	0.4990	0.4600	0.4208	0.4944
153	0.5003	0.4579	0.4209	0.4925
183	0.5000	0.4552	0.4215	0.4882
213	0.5018	0.4524	0.4235	0.4835
243	0.5064	0.4508	0.4245	0.4778
273	0.5103	0.4441	0.4292	0.4758
303	0.5168	0.4374	0.4335	0.4716
333	0.5306	0.4296	0.4391	0.4629
363	0.5403	0.4173	0.4455	0.4541
393	0.5498	0.4098	0.4411	0.4373
423	0.5547	0.4037	0.4422	0.4317
453	0.5595	0.4026	0.4423	0.4188
483	0.5861	0.4089	0.4402	0.4017
513	0.5775	0.4089	0.4334	0.3914
543	0.5656	0.4013	0.4193	0.3765
573	0.5656	0.4013	0.4079	0.3604

To quantify the MOF-based LTs behavior, the luminescence intensity ratios (LIR) between two major emission peaks at 614 nm (due to the ^5^D_0_–^7^F_2_ transitions of Eu^3+^) and 545 nm (due to the ^5^D_4_–^7^F_5_ transitions of Tb^3+^) were determined according to the following eqn (4):^6^4
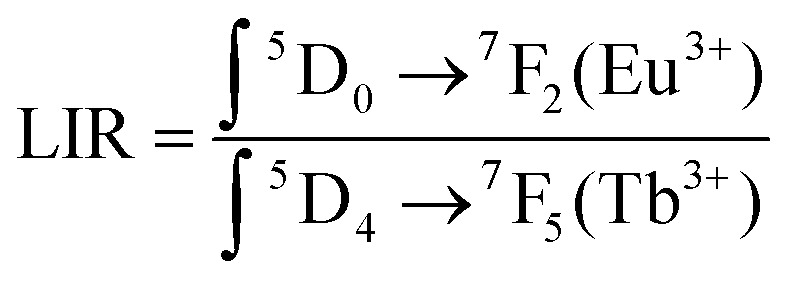


With an increase in temperature from 133 K to 573 K an increase in LIR is observed in both materials, however, the rate of changes was already dependent on the analyzed material. In MOFs without Y^3+^ doping, thermally induced increase of LIR ratio by over 4 times from 1.097 to 4.902 was observed, while addition of Y^3+^ dopant caused nearly 5 times increase in LIR ratio from 0.447 to 2.215.

**Fig. 7:**
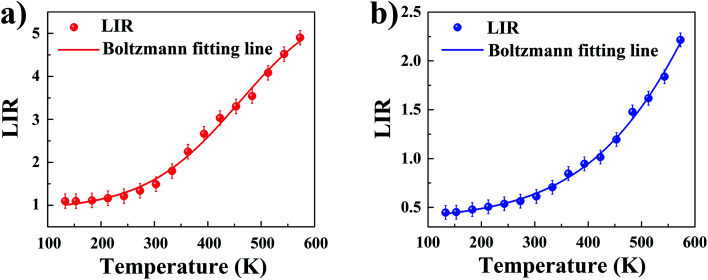
The luminescence intensity ratio (LIR) *versus* temperature of the nanothermometers: Zr-BDC:Eu^3+^,Tb^3+^ (a) and YZ-BDC:Eu^3+^,Tb^3+^ (b). Where the experimental data is marked as points and the solid line corresponds to the Boltzmann fitting results.

The LIR dependence plots of Zr-BDC:Eu^3+^,Tb^3+^ and YZ-BDC:Eu^3+^,Tb^3+^ along with the fitting curves using the Boltzmann equation (eqn (5)) are shown in Fig. 7a and b, respectively.5
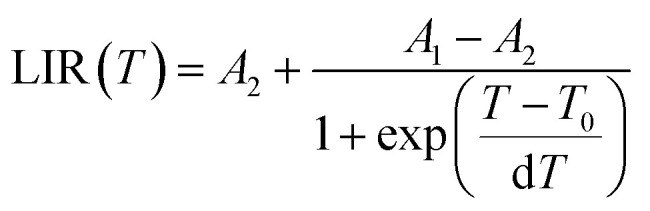


In which, the *A*_1_, *A*_2_, *T*_0_ and d*T* parameters collected by fitting parameters of Boltzmann equation.^25^

The obtained results also show that the presence of Y^3+^ ions not only allows tuning the emission colors from red to yellow, but also can be used to change the LIR values of YZ-BDC:Eu^3+^,Tb^3+^ nanothermometers.

**Fig. 8:**
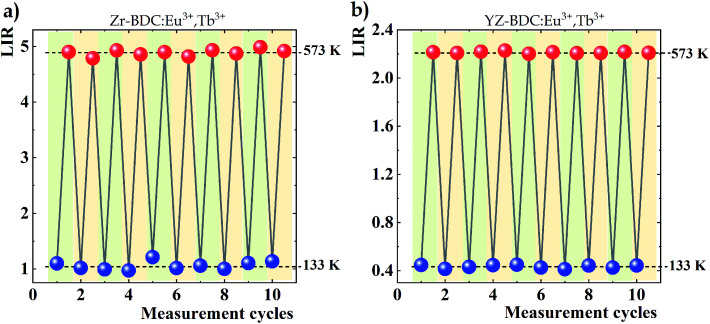
Changes in LIR values over 10 heating and cooling cycles between the extremes of the temperature range analysed obtained for Zr-BDC:Eu^3+^,Tb^3+^ (a) and YZ-BDC:Eu^3+^,Tb^3+^ (b).

Variations in the temperature-dependent LIR parameter values were also examined for repeatability of results. Ten heating and cooling cycles were carried out from the lowest to the highest analyzed temperature, *i.e.* from 133 K to 573 K, respectively (Fig. 8). Although satisfactory repeatability of LIR parameter value changes is obtained for both analyzed phosphors, deviations from a constant value are evident in the absence of doping with Y^3+^ ions, especially visible in the case of measurements at 133 K, while an almost constant value is observed in YZ-BDC:Eu^3+^,Tb^3+^ nanothermometers. The obtained results thus highlight another positive aspect of doping with Y^3+^ ions on the thermal stability of the materials.

To evaluate the performance of YZ-BDC:Eu^3+^,Tb^3+^ nanothermometers compared to Zr-BDC:Eu^3+^,Tb^3+^ nanothermometers without the presence of Y^3+^ ions, the absolute sensitivity (*S*_A_) and relative sensitivity (*S*_R_) to temperature changes were calculated according to eqn (6) and (7), respectively.^6^6
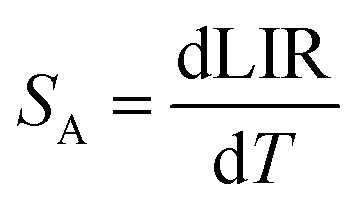
7
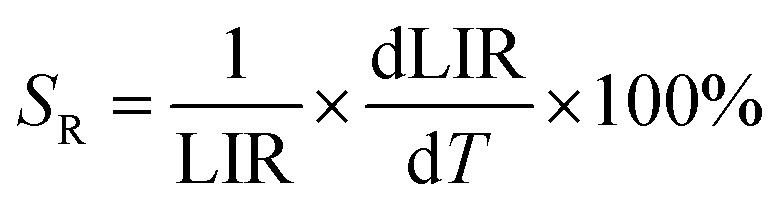


**Fig. 9:**
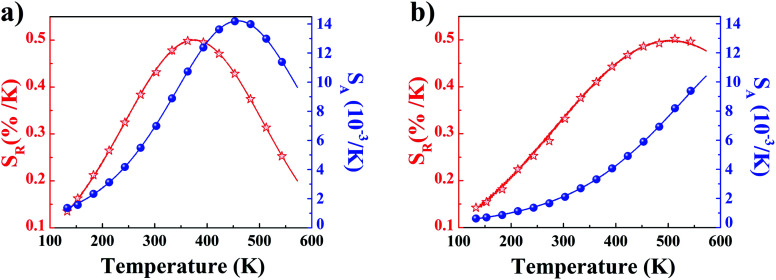
The temperature dependence of the absolute sensitivity (*S*_A_, blue line) and relative sensitivity (*S*_R_, red line) of the Zr-BDC:Eu^3+^,Tb^3+^ (a) and YZ-BDC:Eu^3+^,Tb^3+^ (b).

The temperature dependencies of absolute and relative sensitivity of YZ-BDC:Eu^3+^,Tb^3+^ nanothermometers compared to Zr-BDC:Eu^3+^,Tb^3+^ without the presence of Y^3+^ ions are shown in Fig. 9. As can be seen, both absolute and relative sensitivity values show a similar trend of change and increase with increasing temperature up to a certain critical temperature. In Zr-BDC:Eu^3+^,Tb^3+^ nanothermometers without the presence of Y^3+^, the absolute sensitivity values increased monotonically up to a temperature of 480 K, after which their value began to decrease (Fig. 9a), while in YZ-BDC:Eu^3+^,Tb^3+^ nanothermometers, an increase in the absolute sensitivity values was observed over the entire temperature range analyzed (Fig. 9b).

As shown in Fig. 9a, for Zr-BDC:Eu^3+^,Tb^3+^ nanothermometers without the presence of Y^3+^ ions, the maximum relative sensitivity value was *S*_R_ = 0.501%/K and this was reached at 378 K. In this materials nonmonotonic variation of *S*_R_ value as a function of temperature was observed and at temperatures above 378 K a decrease in the sensitivity value up to 0.200 at 573 K was observed. Although no significant improvement in the maximum relative sensitivity value (*S*_R_ = 0.502%/K) was noted for the YZ-BDC:Eu^3+^,Tb^3+^ nanothermometers, a significant shift of the maximum relative sensitivity towards higher temperatures up to a temperature of 513 K was recorded (Fig. 9b). Beyond this temperature, the relative sensitivity of the thermometer decreased slightly reaching 0.476%/K at 573 K. Based on the analysis of the above results, it can be concluded that by consciously doping the materials with a small amount of Y^3+^ ions, it is possible to intentionally shift the maximum sensitivity to higher temperatures and thus widen the useful temperature range. The obtained results confirmed that the presence of Y^3+^ ions contributes to the increased thermal stability and widens the useful temperature range thus confirming the potential of the YZ-BDC:Eu^3+^,Tb^3+^ nanothermometers for thermal sensing over a wide range of temperatures from near 100 K to high temperatures (reaching > 510 K).

The results obtained were also compared with other thermometers based on MOFs or other amorphous materials (Table 2). Among the works analyzed, it can be observed that the working mode with the maximal sensitivity of most of these thermometers falls rather in the low temperature range from 200 to 350 K, while the maximal sensitivity of the Zr-BDC:Tb^3+^,Eu^3+^ nanothermometer presented in this work covers the temperature range above 500 K enabling its implementation in applications requiring such high temperatures. Furthermore, among the phosphors based on the luminescence of Tb^3+^ and Eu^3+^ ion pairs, the thermometer presented in this work shows one of the higher relative sensitivities, confirming its superiority over other thermometers of this type.

**Table 2:** Comparison of luminescent thermometers based on MOFs and other amorphous materials

Host matrix	Luminescent ion	Relative sensitivity (%/K)	Maximum sensitivity temperature	Publication
[Tb_0.99_Eu_0.01_(hfa)_3_(dpbp)]_n_	Tb^3+^–Eu^3+^	0.52	200	28
Silicate glass	Yb^3+^–Er^3+^	0.33	296	29
PKAZLFEr glasses	Er^3+^	0.13	298	30
Ba_2_NaNb_5_O_15_ glass-ceramics	Yb^3+^–Er^3+^	0.9	298	31
		1.19		
Calcium yttrium silicate (CYS) ceramic powder	Nd^3+^	1.59	298	32
Tb_0.9_Eu_0.1_L	Tb^3+^–Eu^3+^	0.11	300	33
Tb_0.8_Eu_0.2_L		0.15		
Tb_0.7_Eu_0.3_L		0.17		
Ba_4_Y_3_F_17_ glass ceramics	Yb^3+^–Er^3+^	1.13	300	34
TeO_2_–B_2_O_3_ (TBO) glasses	Yb^3+^–Er^3+^	0.73	313	35
Tb_0.99_Eu_0.01_(BDC)_1.5_(H_2_O)_2_ MOF	Tb^3+^–Eu^3+^	0.31	318	36
Chalcogenide glass	Yb^3+^–Er^3+^	0.52	443	37
Tellurite glass	Yb^3+^–Er^3+^	0.53	473	38
β-NaGdF_4_ glass ceramics	Yb^3+^–Er^3+^	0.37	580	39
Zr-BDC	Tb^3+^–Eu^3+^	0.5	378	This work
YZ-BDC			513	

## 4. Conclusions

In summary, the YZ-BDC:Eu^3+^,Tb^3+^ nanothermometers with chemical formula is Zr_1−*x*_OC_6_H_4_(COO)_2_:RE^3+^ (RE = Eu, Tb, Y) were successfully synthesized using the microwave reactor at 120 °C for 15 min. The synthesized YZ-BDC:Eu^3+^,Tb^3+^ nanothermometers with sizes of about 10–30 nm exhibit high thermal stability (>660 K) and amorphous structure, with multiple BET surface area, total pore volume and average pore diameter of 530 m^2^ g^−1^, 0.45 cm^3^ g^−1^, and 3.4 nm, respectively. The temperature-dependent emission spectra of synthesized YZ-BDC:Eu^3+^,Tb^3+^ nanothermometers compared with Zr-BDC:Eu^3+^,Tb^3+^ nanothermometers without the presence of Y^3+^ ions were investigated over a wide temperature range from 133 to 573 K. The results showed that both the temperature and the presence of Y^3+^ ions in the host significantly affected the relative intensity of the band at 614 nm originating from Eu^3+^ ions and the band at 545 nm associated with the luminescence of Tb^3+^ ions. As a result, a different impact of temperature on emission color changes was noted in the two materials analyzed. Zr-BDC:Eu^3+^,Tb^3+^ nanothermometers without the presence of Y^3+^ ions, the emission color changed from orange to red, while in YZ-BDC:Eu^3+^,Tb^3+^ nanothermometers, an increase in temperature from 133 to 573 K resulted in change of emission color from yellow to orange. Although there was no noticeable impact of material on the maximum relative sensitivity, in the material doped with Y^3+^ ions, a significant shift in the temperature at which the maximum relative sensitivity occurred towards higher temperatures was observed (from 378 K in the material without Y^3+^ ion doping to 513 K when co-doped). The results show that by doping with a small amount of Y^3+^ ions, it is possible to improve the thermal stability of materials and modulate the temperature range with high sensitivity.

## Author contributions

Lam Thi Kieu Giang performed conceptualization, resources, data curation, writing – original draft preparation, project administration, funding acquisition. Karolina Trejgis and Łukasz Marciniak performed investigation of thermal properties, methodology for temperature dependent measurement, data curation, writing – review and editing, conceptualization. Agnieszka Opalińska performed investigation and formal analysis of (BET) surface area. Iwona E. Koltsov performed investigation and formal analysis of FTIR and STA analysis, review and editing. Witold Łojkowski performed supervision, funding acquisition.

## Conflicts of interest

There are no conflicts to declare.

## Supplementary Material
