# Correction: Synthesis and characterizations of YZ-BDC:Eu^3+^,Tb^3+^ nanothermometers for luminescence-based temperature sensing

**DOI:** 10.1039/d2ra90049a

**Published:** 2022-05-16

**Authors:** Lam Thi Kieu Giang, Karolina Trejgis, Łukasz Marciniak, Agnieszka Opalińska, Iwona E. Koltsov, Witold Łojkowski

**Affiliations:** Institute of Materials Science, Vietnam Academy of Science and Technology 18 Hoang Quoc Viet, Cau Giay Hanoi Vietnam giangltk@ims.vast.ac.vn; Graduate University of Science and Technology, Vietnam Academy of Science and Technology 18, Hoang Quoc Viet, Cau Giay Hanoi Vietnam; Institute of Low Temperature and Structural Research, Polish Academy of Sciences Okólna 2 50-422 Wrocław Poland; Institute of High Pressure Physics, Polish Academy of Sciences Sokolowska Street 29/37 Warsaw Poland

## Abstract

Correction for ‘Synthesis and characterizations of YZ-BDC:Eu^3+^,Tb^3+^ nanothermometers for luminescence-based temperature sensing’ by Lam Thi Kieu Giang *et al.*, *RSC Adv.*, 2022, **12**, 13065–13073, https://doi.org/10.1039/D2RA01759H.

The authors regret the omission of a funding acknowledgement in the original article. This acknowledgement is given below.

Karolina Trejgis is supported by the Foundation for Polish Science (FNP).

The Royal Society of Chemistry apologises for these errors and any consequent inconvenience to authors and readers.

## Supplementary Material

